# Anonymizing at-home fitness: enhancing privacy and motivation with virtual reality and try-on

**DOI:** 10.3389/fpubh.2023.1333776

**Published:** 2023-12-19

**Authors:** Kang-Il Yoon, Tae-Soo Jeong, Seung-Chan Kim, Soo-Chul Lim

**Affiliations:** ^1^Department of Mechanical, Robotics, and Energy Engineering, Dongguk University, Seoul, Republic of Korea; ^2^Machine Learning Systems Lab, College of Sports Science, Sungkyunkwan University, Suwon, Republic of Korea

**Keywords:** smart applications, fitness apps, virtual reality exercise, virtual try-on, physical health, physical activity

## Abstract

**Introduction:**

This study aimed to address privacy concerns associated with video conferencing tools used in home-based exercise training. To that end, a method that could anonymize participants' appearances and exercise environments during at-home fitness sessions was proposed.

**Methods:**

This method combines virtual reality for 3-D human-model rendering using key-points tracking with a virtual try-on system enhanced by UV mapping and instance segmentation. To validate the proposed method, we conducted a user study by recruiting participants to assess effectiveness of virtual reality and virtual try-on in terms of privacy protection, self-confidence, and coaching satisfaction.

**Results:**

Experimental results demonstrated the effectiveness and improved user experience of using virtual reality or virtual try-on in remote fitness, particularly in enhancing privacy protection and self-confidence with statistical significance. However, no significant differences were noted in coaching satisfaction.

**Discussion:**

These findings confirmed the efficacy of our proposed approach. We believe that the proposed approach can significantly contribute to the future of remote fitness training, offering a more secure and engaging environment for users, thereby potentially increasing adherence to fitness regimens and overall physical wellbeing.

## 1 Introduction

Exercise can improve physical appearance. It is also widely recognized as an essential factor for enhancing and maintaining health in the long term. However, the proportion of individuals engaging in insufficient physical activity remains high worldwide, rendering them more susceptible to non-communicable diseases (1, 2). Furthermore, people in most countries faced restrictions of physical activity during the COVID-19 pandemic, resulting in significant reduction of overall physical activity levels (3–5). Although these restrictions have started to ease, physical activity levels of people have not recovered fully (6, 7).

In light of these concerns, home-based exercise programs have gained attention as potential alternatives as they can improve accessibility and save time. Home-based exercise is a useful solution for busy individuals who struggle to balance work and physical activity. For instance, home-based exercise has already been proven to be effective for various groups, such as female caregivers with limited time for exercise (8), frail individuals (9), and geriatric patients with cognitive impairments (10). In response to this demand, various online platforms have emerged and shared exercise videos that can be followed at home, benefiting many users. There is evidence that patients who undergo training using recorded exercise videos following COVID-19 hospitalization show encouraging cardiovascular, respiratory, and functional outcomes (11). In addition, home-based exercises can provide clinical benefits to individuals with severe SARS-CoV-2 infection (12). However, participants in these home-based exercise programs show a tendency to experience difficulty when first attempting such exercises.

Video conferencing technology has gained attention in home-based exercise training as it can provide real-time feedback coaching and supervised exercise programs. Exercise training using video conferencing can also provide motivation and offer personalized exercise programs that overcome distance and time constraints. Online exercise programs can provide guidance for independent workouts and monitor continuous exercise, enabling individuals to exercise conveniently from their own residences. Moreover, exercise becomes possible anytime and anywhere with internet connectivity, thus greatly mitigating limitations of time and location.

Home-based exercise training programs often rely on screen sharing for delivering exercise instructions. However, due to privacy concerns, this method may not be considered ideal. This is because exercise videos can contain personal information and sharing videos from home-based training sessions could potentially lead to unintended exposure of such details. In light of the growing popularity of video conferencing tools, recent research has focused on understanding vulnerabilities and risks associated with data privacy in this context (13, 14). Furthermore, other recent studies (15, 16) have highlighted privacy concerns among participants in home-based fitness training, which can lead to reluctance in video sharing.

Individuals participating in home-based exercise training often need to expose their clothing and appearance to others, potentially evoking Social Physique Anxiety (SPA). SPA is an emotional response that arises from concerns about others scrutinizing or judging their physical appearance (17). This anxiety is often triggered when individuals feel their physique does not match societal ideals of an “ideal body” (17). Elevated levels of SPA often correlate with lower adherence to physical activity and decreased participation rates in such programs (18). These individual psychological factors, including privacy concerns, social pressure, and self-consciousness, can make exercise participants hesitant to share their videos, with many preferring not to share their videos for these reasons (16). However, interventions aimed at reducing SPA might significantly enhance motivation and promote behaviors that encourage physical activity (17). Additionally, the concept of self-presentational efficacy (SPE) expectancy, defined as a subjective likelihood of successfully of conveying desired impressions to others, has been identified as a key factor influencing both exercise behavior and SPA (19). Elevating SPE expectancy could potentially be an effective strategy to encourage engagement in physical activity. It directly addresses SPA challenges in home-based exercise programs and aligns with findings that link SPA and SPE to physical activity participation (20).

In support of these findings, Lee et al. (21) have provided compelling evidence on benefits of using avatars in video conferencing. Their study demonstrated that avatars, rather than actual appearances, could enhance participant confidence and engagement. Their findings reveal that using avatars can foster better interactions, alleviate anxiety about appearances and behaviors, and promote increased concentration and willingness to engage in virtual communication. Similarly, observable changes in physical activity (PA) have been noted in virtual environments based on the selection of avatars. For example, it has been found that overweight children randomly allocated to avatars depicting a typical body size demonstrate enhanced performance in a PA-centric video game (22). This cohort also displayed heightened exercise motivation and a more favorable disposition toward PA compared to their counterparts assigned to obese avatars. Likewise, women assigned to slender avatars during engagement in a tennis exergame exhibited heightened PA compared to those assigned to obese avatars (23). These findings are reproducible within men cohorts, wherein an association exists between increased PA and allocation to slim rather than overweight avatars (24). This aligns with the notion that modifying virtual self-representation, such as through avatars, could be an innovative approach to manage SPA and improve exercise adherence. From this perspective, this paper presents a method to explore potential solutions for promoting exercise participation and addressing privacy concerns associated with home-based exercise training.

This study primarily focused on privacy concerns associated with video conference technology used in home-based exercise training programs. Additionally, we aimed to improve privacy protection while exploring approaches to reduce the burden caused by exposing the exercise environment and process, with the goal of enhancing exercise motivation. To achieve this, we first identified vulnerabilities of current video conference technologies used and proposed alternatives to address privacy concerns. Furthermore, we identified factors contributing to perceived burden in an exercise environment and process to suggest mitigation strategies. By doing so, we seek to find safe and effective methods to utilize video conference technologies in home-based exercise training while simultaneously ensuring privacy protection and promoting user engagement during exercise.

This study used a novel approach that could leverage VR technology (3D human-model rendering based on 3D key-points tracking) and virtual try-on network (UV mapping-enhanced instance-segmentation-based virtual try-on network: UVI-VTON) to mitigate constraints associated with user privacy protection effectively while alleviating the burden experienced by users in the context of screen sharing for educational purposes. The remainder of this manuscript is organized as follows. Section 2 presents a review of related research from perspectives of VR-based conference and VR-based exercise. Section 3 introduces applications of VR and UVI-VTON in home-based exercise training. Section 4 presents an empirical study evaluating the usability of the proposed approach. Sections 5 describes statistical analysis results and discussion of the usability survey. Section 6 then summarizes key findings of this study and suggests some future research directions.

## 2 Related work

This study was inspired by existing research incorporating VR technologies, which could be broadly categorized into two groups: VR and VTON.

### 2.1 Virtual reality

Currently, VR technology is gaining attention as a means of promoting and assisting physical activity. The use of VR in exercise has potential to generate more favorable effects on physiological, psychological, and rehabilitative outcomes of individuals than conventional exercise methodologies (25). Prior to the COVID-19 pandemic, these studies were already underway, showcasing the efficacy of virtual reality interventions. For instance, a comparative study between virtual reality game exercises and ball exercises has highlighted superior benefits of VR games in enhancing balance abilities of older adults (26). Arlati et al. have introduced VR-based SocialBike, tailored to improve clinical outcomes in older adults (27). Furthermore, a study focusing on individuals with cardiovascular diseases has demonstrated significant enhancements in body composition, lipid profile, and eating patterns through cardiac rehabilitation within a virtual reality environment in comparison with conventional rehabilitation methods (28).

Since the onset of the COVID-19 pandemic, there has been a resurgence of interest in this research domain. Previous research has indicated that virtual reality exercise and fitness apps can be used as effective strategies for enhancing physical and mental wellbeing during the pandemic period (29). Additionally, during COVID-19 lockdowns, empirical studies have demonstrated that the use of smart applications, including live streaming exercise classes and virtual reality fitness programs, has a positive impact by promoting physical activity (30). The necessity of replacing outdoor exercise with a viable alternative has been increasingly emphasized since the COVID-19 outbreak. VR has stood out as a pivotal alternative to outdoor exercise, demonstrating a substantial capacity to improve cognitive function and mitigate motor disabilities (31). In particular, there have been active discussions on the necessity and effectiveness of alternative exercises in academic circles for individuals facing difficulties with outdoor activities, such as older adults (32), patients with post-COVID-19 condition (33), patients recovering from COVID-related pneumonia (34), physically inactive individuals during the COVID-19 lockdown (30), and patients diagnosed with Parkinson's disease (35). In addition, both specialized and gaming VR can be effective in treating upper-extremity impairments, with specialized VR showing potential for improving balance in patients with neurological conditions (36). In contrast to studies focusing on individuals with specific physical limitations, the study by Ng et al. (37) investigated the impact of VR- or augmented reality (AR)-enhanced training on physical activity in healthy individuals through a meta-analysis to. It demonstrated that VR intervention had substantial effects on physical activity levels, minor effects on performance, but no effects on psychological outcomes.

### 2.2 Virtual try-on network

VTONs leverage advanced computer-vision and machine-learning techniques to simulate the process of virtually trying on clothing items. By overlaying virtual garments on a user's body, individuals can visualize how different clothing items would look on them without actually wearing them. Recent advancements in deep-learning-based VTONs have shown significant improvements in clothing synthesis performances. For instance, context-driven virtual try-on network (C-VTON) (38) employs discriminators specific to different types of contextual information, allowing enhanced clothing synthesis quality. The dual-branch collaborative transformer (DBCT) (39) utilizes a transformer-based architecture and cross-modal information to improve the virtual try-on process. To address the alignment of target garments to corresponding body parts, a novel global appearance flow estimation model has been proposed, which warps clothing spatially (40). The Dress Code dataset (41) containing images of diverse categories of clothes has also been introduced. This dataset enables the generation of high-resolution try-on images. These virtual try-on technologies have been utilized for virtual fitting in online shopping. Several studies have investigated their impacts. Research has shown that attitudes of consumers toward virtual try-on can influence their online purchasing behaviors (42). Furthermore, a review of literature has shown a wide range of psychological (cognitive, emotional, and social) and behavioral consequences associated with virtual try-on in the context of shopping (43).

## 3 Methods

The aim of this study was to develop a methodology utilizing deep-learning-based models to safeguard the privacy of individuals engaging in home-based exercises and to encourage exercise participation. Specifically, the methodology consisted of two key features: (1) VR (3D human-model rendering based on 3D key-points tracking); and (2) virtual try-on network (UVI-VTON). The 3D human model rendering was designed for real-time motion tracking and avatar rendering based on images of individuals engaged in movement. It was generated by predicting joint positions of users using deep-learning algorithms and mapping them to a prebuilt human model. The UVI-VTON was developed to enhance video generation speed compared to the conventional virtual try-on network, enabling real-time video generation while improving video quality. It could generate an image of the user wearing different clothing, allowing them to exercise without being self-conscious about their appearance.

To evaluate the effectiveness and usability of this methodology, we conducted experiments and a usability survey. Experiments involved testing performances of deep-learning models used in real-world scenarios. The usability survey involved evaluating user experiences of the methodology, including user satisfaction and feedback assessments on coaching effectiveness from the perspectives of privacy protection and motivation. In this section, we will describe each of these components in detail, including methods used, results obtained, and implications for future development.

### 3.1 VR: 3D human-model rendering

**Figure 1:**
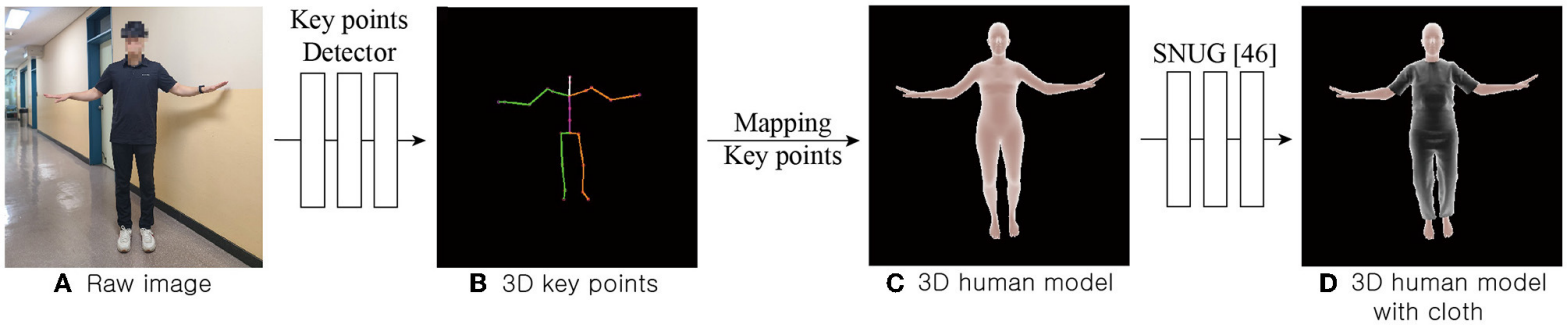
Procedure for 3D human modeling with cloth. Given the raw frame **(A)**, the detected 3D key points **(B)** are mapped to a 3D human model **(C)** constructed using SMPL model (47). Subsequently, the SNUG model (46) is used to apply clothing to the corresponding 3D human model to render a 3D human model with cloth **(D)**.

The skinned multi person linear (SMPL) model widely used in computer vision was employed as the 3D human model to construct human shapes. The SMPL model comprised a 3D body mesh that could transform shapes of the joints and muscles of a person to resemble their actual appearance depending on body shape and key points. The SMPL model could be rendered from estimated parameters based on real-time image frames. Estimated SMPL parameters could then be used with deep-learning-based 3D key-points estimation models. This instance 3D key points estimation model has been discussed in various ways in the field of computer vision. As shown in Figures 1A, B, HRNet was adopted (44) to detect and track 3D key points of a person from a single image frame.

The SMPL model represents the shape of a person without clothing. Using the generated model as an avatar can potentially make users feel uncomfortable and unpleasant. In this research, to create a more comfortable and confident representation of the user, the avatar representing the user tried on clothing. Furthermore, various clothing designs were implemented with the ability for color selection to reflect individuality of the user to achieve effects discussed in a previous study (45). This demonstrate that increased avatar personalization can lead to greater body ownership, presence, and emotional responses. As shown in Figures 1C, D, the SNUG model (46) was adopted to try on clothes over vertices of the SMPL mesh. This model utilizes physical loss to depict dynamic aspects of clothing by taking into account movement speed of the human body, resulting in a more realistic representation of garments. Three types of top and two types of bottom apparel were applied to the SMPL model (47) using this approach.

### 3.2 UVI-VTON: user image regenerated with reference garments using generative adversarial network

**Figure 2:**
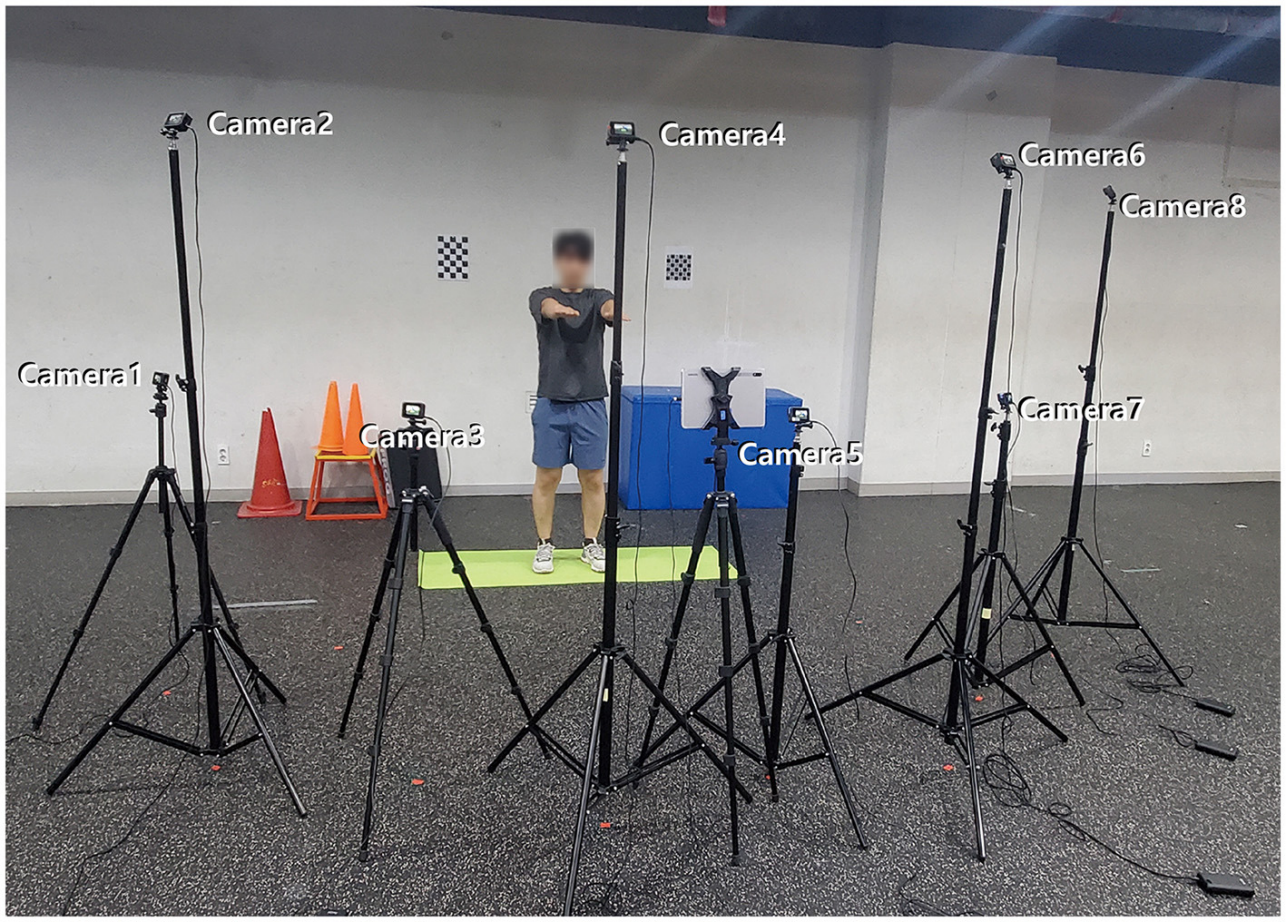
Experimental setup with eight installed cameras that are situated at distinct angles and oriented toward the subject.

For training the UVI-VTON, we captured movements of subjects performing yoga and weight training. These two exercise types were used in our home-based training application. One experimental group composed of 14 males and 7 females who performed weight training. Another experimental group consisted of 6 males and 14 females engaged in yoga, resulting in the collection of exercise videos from a total of 41 participants. Each participant executed 10 prescribed movements specified in Table 1 for a single exercise category, i.e., either yoga or weight training. For each exercise movement, participants alternated between wearing four sets of upper- and lower-body clothing and performed exercise movements iteratively. This process resulted in a total of 40 sets of exercises (1 exercise category × 10 movements × 4 clothing sets) performed by each participant. As shown in Figure 2, these scenes were captured using a setup of eight synchronized cameras (GoPro Hero5) positioned to face the subject, with each video recorded at 30 frames per second. The resulting dataset contained a total of 20,606,496 image frames (8 cameras × 2,575,812 image frames extracted from the exercise videos of 41 people) that captured activities of the 41 participants across the 8 cameras. This dataset was used to train the model.

**Table 1:** Order of exercises in home-based exercise training.

	**Week**	**Training poses**
Weight training	Week 1	Squat—Lunge—Push-up—Bird Dog—Plank
	Week 2	Bridge—Shoulder Press—Side Lateral Raise—Side Plank—Mountain Climber
	Week 3	Push-up—Plank—Shoulder Press—Squat—Side Plank
	Week 4	Side Lateral Raise—Lunge—Bridge—Bird Dog—Mountain Climber
Yoga	Week 5	Seated Forward Bend Pose—Half Moon Pose—Triangle Pose—Warrior 2—Cat Cow Pose
	Week 6	Half Moon Pose—Pelvic Opening—Warrior 1—Warrior 2—Triangle Pose—Chair Pose—Side Plank—Cobra Pose
	Week 7	Half Moon Pose—Triangle Pose—Chair Pose—Warrior 2—Cobra Pose
	Week 8	Seated Forward Bend Pose—Cat Cow Pose—Pelvic Opening—Warrior 1—Side Plank

UVI-VTON consisted of three main components: a segmentation predictor, a try-on network for the top (TON-T), and a try-on network for the bottom (TON-B). The segmentation predictor generated a segmentation image from the raw image, where areas for wearing top and bottom garments were indicated explicitly. This segmentation image was utilized by the two try-on networks to try on respective clothing items.

**Figure 3:**
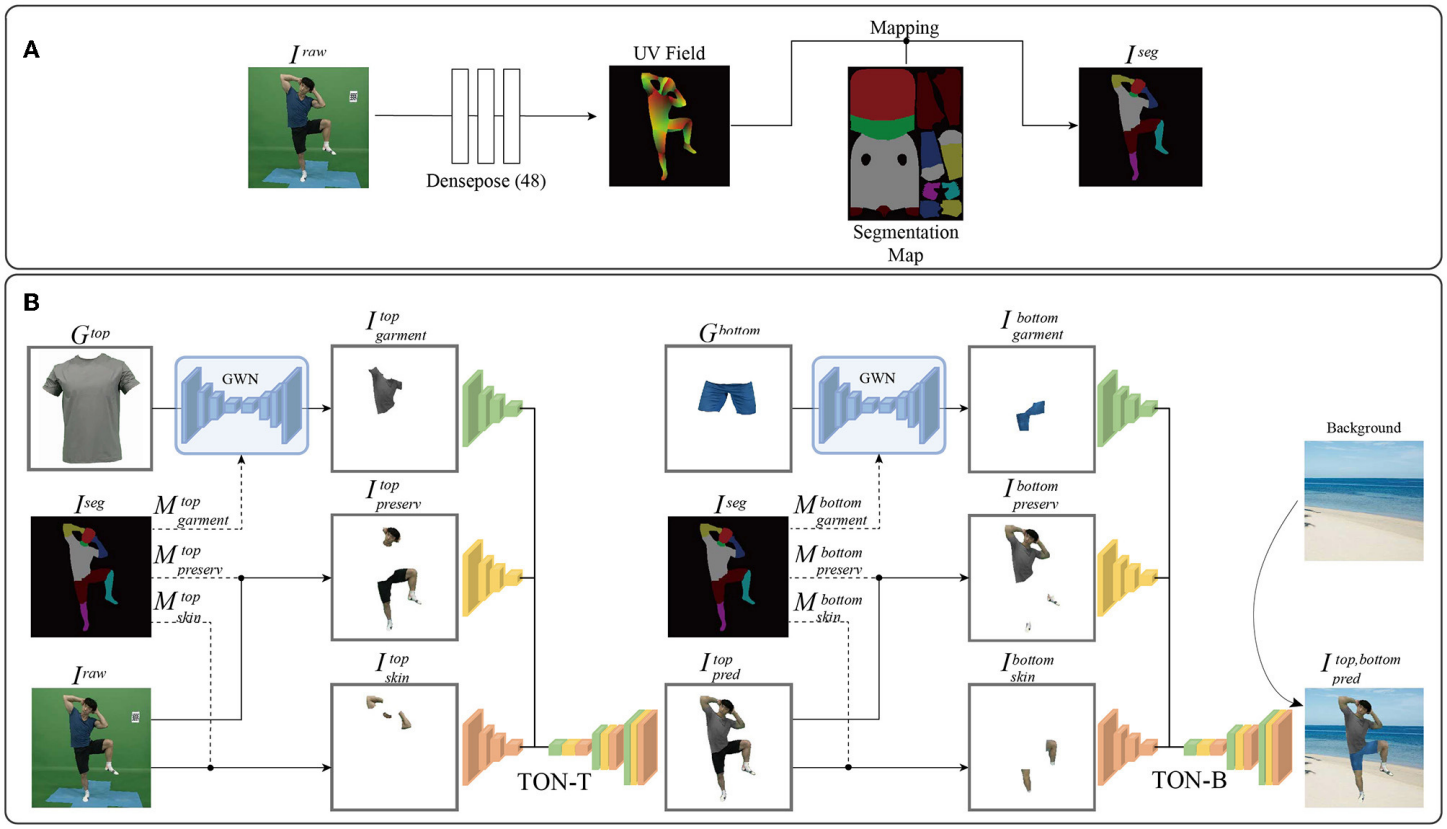
**(A)** Segmentation predictor: It aims to generate a semantic segmentation image (*I*^*seg*^) in real time from a raw image (*I*^*raw*^) by applying the segmentation map to the UV field predicted by Densepose (48). **(B)** Overview of the proposed network: The semantic segmentation image (*I*^*seg*^) is used to try *G*^*top*^ on *I*^*raw*^, where the garment warping network (GWN) warps *G*^*top*^ to $I_{garment}^{top}$ and inputs it to TON-T. Then, *I*^*raw*^ is divided into the parts for preservation ($I_{preserv}^{top}$) and skin parts for regeneration ($I_{skin}^{top}$), which are used as inputs to TON-T to generate $I_{pred}^{top}$. Similarly, *I*^*seg*^ is employed to try *G*^*bottom*^ on $I_{pred}^{top}$, where the GWN warps *G*^*bottom*^ to $I_{garment}^{bottom}$ and inputs it to TON-T. Subsequently, $I_{pred}^{top}$ is divided into the parts for preservation ($I_{preserv}^{bottom}$) and skin parts for regeneration ($I_{skin}^{bottom}$), which are used as inputs to TON-B to generate $I_{pred}^{top,\;bottom}$.

As shown in Figure 3A, the UV field was extracted from the raw image (*I*^*raw*^) using Densepose (48), enabling pixel-level recognition of human body parts in the image and estimation of their 3D positions and orientations, which were then visualized as density maps. Subsequently, the segmentation predictor applied segmentation maps to this UV field, generating a segmentation image *I*^*seg*^) that delineated a total of 12 distinct body parts: top, bottom, head, neck, left arm, right arm, left hand, right hand, left leg, right leg, left foot, and right foot.

As shown in Figure 3B, the top garment (*G*^*top*^) was processed through the garment warping network (GWN), resulting in a warped top-garment image ($I_{garment}^{top}$) that was fed to TON-T. Additionally, the raw image (*I*^*raw*^) was segmented to produce the semantic segmentation image (*I*^*seg*^), generating parts for preservation ($I_{preserv}^{top}$) and skin parts for regeneration ($I_{skin}^{top}$) in the process of trying on the top. Both these images were used as inputs to TON-T. Applied inputs were then synthesized through TON-T, resulting in the predicted top-replaced image ($I_{pred}^{top}$). This process could simulate a person trying on the top garment to produce $I_{pred}^{top}$ wherein the person appeared to be wearing the replaced top.

To try on *G*^*top*^, semantic segmentation was utilized and divided into three masks: a mask for *G*^*top*^ ($M_{garment}^{top}$), a mask for upper-body skin ($M_{skin}^{top}$) consisting of both arms and neck, and a preservation mask ($M_{preserv}^{top}$) consisting of the bottom, head, both hands, both legs, and both feet. The GWN was used to warp *G*^*top*^ to match the shape of $M_{garment}^{top}$, resulting in $I_{garment}^{top}$. *I*^*raw*^ was divided into preserved parts ($I_{preserv}^{top}$) determined by $M_{preserv}^{top}$ and regenerated skin parts ($I_{skin}^{top}$) of the upper body defined by $M_{skin}^{top}$. Consequently, $I_{preserv}^{top}$, $I_{skin}^{top}$, and $I_{garment}^{top}$ were combined as inputs to TON-T to predict $I_{pred}^{top}.$

TON-B had the same pipeline as TON-T, with the only difference being the reference garments (top or bottom) that users tried on. Similarly, the bottom garment (*G*^*bottom*^) was processed through the GWN, resulting in a warped bottom-garment image ($I_{garment}^{bottom}$) that was then fed to TON-B. Additionally, $I_{pred}^{top}$ was segmented by *I*^*seg*^ to generate parts for preservation ($I_{preserv}^{bottom}$) and skin parts for regeneration ($I_{skin}^{bottom}$) in the process of trying on the bottom. Both these images were then used as inputs to TON-B. These inputs were then synthesized through TON-B, resulting in the predicted top-and-bottom-replaced image ($I_{pred}^{top,\;bottom}$). This process simulated a person trying on the top and bottom garments, resulting in $I_{pred}^{top,\;bottom}$ wherein the person appeared to be wearing the replaced top and bottom apparel.

To try on *G*^*bottom*^, semantic segmentation was utilized and divided into three masks: a mask for *G*^*bottom*^ ($M_{garment}^{bottom}$), a mask for lower-body skin ($M_{skin}^{bottom}$) consisting of both legs, and a preservation mask ($M_{preserv}^{bottom}$) consisting of the top, head, both arms, both hands, and both feet. The GWN was then used to warp *G*^*bottom*^ to match the shape of $M_{garment}^{bottom}$, resulting in $I_{garment}^{top,\;bottom}$. This $I_{pred}^{top}$ was then divided into preserved parts ($I_{preserv}^{bottom}$) determined by $M_{preserv}^{bottom}$ and regenerated skin parts ($I_{skin}^{bottom}$) of the lower body defined by $M_{skin}^{bottom}$. Consequently, $I_{preserv}^{bottom}$, $I_{skin}^{bottom}$, and $I_{garment}^{bottom}$ were combined as inputs to TON-B to predict $I_{pred}^{bottom}$.

To train the proposed network, an objective loss function was constructed by combining four loss components: L2 loss, VGG19-based perceptual loss, second-order smooth constraint loss (49), and adversarial loss computed using the pix2pixHD discriminator (49).

## 4 Experiments

To assess the effectiveness of UVI-VTON and VR, participants were recruited to perform and evaluate home-based exercise training programs using the developed application. Test exercises consisted of weight training and yoga guided by a coach such that participants had the opportunity to try out proposed methodologies instructed by the coach. After completing the exercise session, participants responded to a usability survey.

### 4.1 Participants

Experiments to test UVI-VTON and VR comprised a total of 35 participants, including 5 males and 30 females. These participants aged from 18 to 48 years, with a mean age of 30 (±8) years. Participants were chosen based on self-selection through recruitment advertisements. All participants were enrolled after this study obtained Institutional Review Board (IRB) approval (Sungkyunkwan University IRB approval number: SKKU 2021-12-014).

### 4.2 Procedures

Participants were assigned to five different teams, each having distinct time schedules. Participants joined teams based on their preferred time slots for the experiment. Simultaneously, efforts were made to minimize disparities in team sizes, aiming to achieve a balanced distribution of members among teams. Each team had six to eight members. Experiments were conducted once a week for 8 weeks. During each session, participants engaged in a total of eight exercise sessions, alternating between four sessions each of weight training and yoga, each lasting 30 min. Two coaches, one specializing in yoga and the other in weight training, participated in the experiment. To enhance experiences of participants in home-based exercise training, types and order of exercises were varied over a 4-week period as depicted in Table 1.

**Figure 4:**
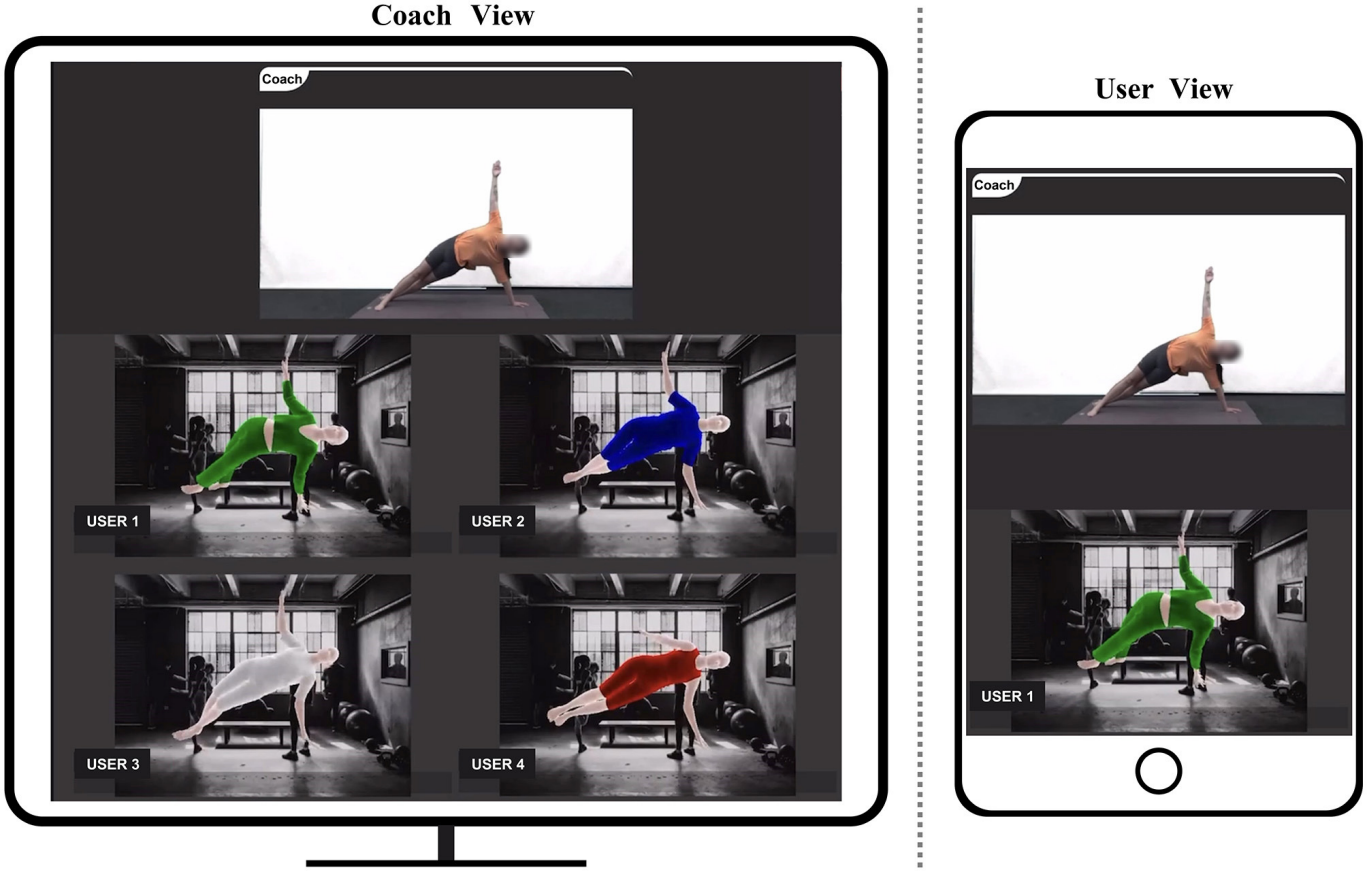
Home-based exercise training with VR (3D human-model rendering based on 3D key-points tracking).

**Figure 5:**
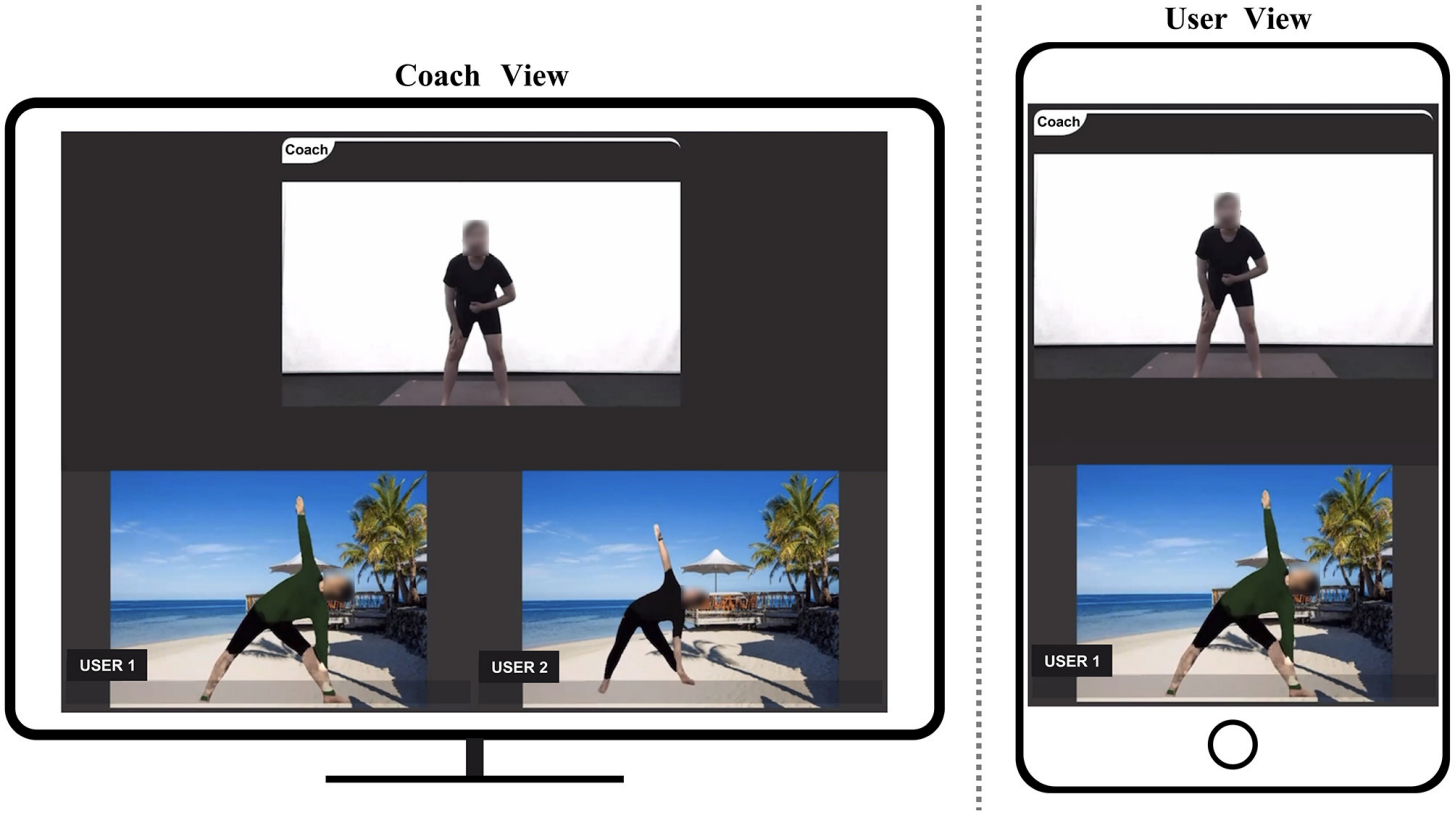
Home-based exercise training with UVI-VTON (UV mapping-enhanced instance-segmentation-based virtual try-on network).

The coach, situated in a prepared studio, shared their own video feed with participants through a camera, while participants shared their video feeds using their mobile devices from the comfort of their homes. The coach provided instructions for prescribed exercises in a sequential manner. Upon demonstrating each exercise, participants repeated movements accordingly. Simultaneously, participants adjusted their exercise postures in accordance with instructions from the coach, allowing for personalized training modifications. To assess the impact of utilizing UVI-VTON and VR in home-based exercise training compared to not using any methodology, participants engaged in training sessions employing UVI-VTON and VR as well as sessions where no specific methodology was employed. As shown in Figures 4, 5, when utilizing methodologies, participants activated either UVI-VTON or VR upon request from the coach, thereby sharing their real-time modified visual representations on the screen.

### 4.3 Usability survey

Participants were asked to respond to a survey based on their own experiences with home-based exercise training after an 8-week period of participation. As depicted in Table 2, the usability survey was categorized under three evaluation factors: privacy protection scale (PPS), self-confidence and motivation scale (SMS), and coaching satisfaction scale (CSS). The PPS questionnaire comprehensively assessed participants' perceived burden of camera recording and the extent to which their privacy was protected. This survey comprised three items rated on a scale of 1 (strongly disagree) to 5 (strongly agree) by participants. The SMS questionnaire measures self-confidence and motivation effects derived from home-based exercise training. This questionnaire consisted of two items rated on a scale of 1 (strongly disagree) to 5 (strongly agree) by participants. The CSS questionnaire measures participants' perceptions of whether their exercise performances were accurately conveyed to the instructor and whether they received appropriate feedback based on their experiences in home-based exercise training. This questionnaire comprised two items rated on a scale of 1 (strongly disagree) to 5 (strongly agree) by participants. The raw data of usability survey is depicted in Table 3. Usability survey data were analyzed using one-way analysis of variance (ANOVA). Significant interactions and main effects were investigated through Bonferroni-corrected pairwise comparisons. A significance level of *p* < 0.05 was considered to be statistically significant. Precise values of *p* are reported unless *p* < 0.001. Descriptive statistics for all data are displayed as mean ± standard deviation (SD) with 95% confidence intervals (CIs).

**Table 2:** Survey questions administered upon completion of home-based exercise training.

	**Question**
Privacy Protection Scale (PPS)	1. Is the user privacy protected effectively during home-based exercise training?
	2. Is the user privacy adequately ensured during home-based exercise training?
	3. Is there no burden associated with camera recording for home training purposes?
Self-confidence and Motivation Scale (SMS)	4. Did you feel confident during the exercise?
	5. Did you feel motivated to exercise?
Coaching Satisfaction Scale (CSS)	6. Are you satisfied with the quality of the instructions from the coach for your exercise posture and feedback?

**Table 3:** Raw data of usability survey (mean ± SD).

	**PPS**			**SMS**		**CSS**
	**Question 1**	**Question 2**	**Question 3**	**Question 4**	**Question 5**	**Question 6**
Use of nothing	4.14 ± 0.97	4.11 ± 0.96	3.63 ± 1.40	3.86 ± 1.22	3.89 ± 1.32	4.71 ± 0.53
Use of UVI-VTON	4.66 ± 0.64	4.71 ± 0.57	4.60 ± 0.74	4.54 ± 0.78	4.63 ± 0.73	4.63 ± 0.84
Use of VR	4.83 ± 0.45	4.86 ± 0.43	4.63 ± 0.73	4.49 ± 0.85	4.49 ± 0.82	4.51 ± 0.85

## 5 Results

Results of the ANOVA conducted for PPS, SMS, and CSS of home-based exercise training with VR or UVI-VTON indicated no significant effects, suggesting that both methodologies elicited similar emotional responses. However, when home-based exercise training with VR or UVI-VTON were compared to home-based exercise training without VR or UVI-VTON, significant differences in PPS, SMS, and CSS were observed.

When conducting home-based exercise training, significant differences were observed for question 1 of PPS among the use of UVI-VTON, VR, and no intervention [*F*_(2, 68)_ = 15.880; *p* < 0.001]. *Post-hoc* analysis revealed no significant difference (*p* = 0.095) between the use of UVI-VTON and VR for question 1. However, the use of UVI-VTON showed a significantly higher rating of 0.514 (*p* = 0.003) than the use of nothing for question 1. The use of VR also exhibited a significantly higher rating of 0.686 (*p* < 0.001) than the use of nothing for question 1.

When conducting home-based exercise training, significant differences were observed in question 2 of PPS among the use of UVI-VTON, VR, and nothing [*F*_(2, 68)_ = 20.035; *p* < 0.001]. *Post-hoc* analysis revealed no significant difference (*p* = 0.069) between the use of UVI-VTON and VR for question 2. However, the use of UVI-VTON showed a significantly higher rating of 0.600 (*p* < 0.001) than the use of noting for question 2. The use of VR also exhibited a significantly higher rating of 0.743 (*p* < 0.001) than the use of nothing for question 2.

When conducting home-based exercise training, significant differences were observed in question 3 of PPS among the use of UVI-VTON, VR, and nothing [*F*_(2, 68)_ = 16.302; *p* < 0.001]. *Post-hoc* analysis revealed no significant difference (*p* = 1.000) between the use of UVI-VTON and VR for question 3. However, the use of UVI-VTON showed a significantly higher rating of 0.971 (*p* < 0.001) than the use of nothing for question 3. The use of VR also exhibited a significantly higher rating of 1.000 (*p* < 0.001) than the use of nothing for question 3.

When conducting home-based exercise training, significant differences were observed in question 4 of SMS among the use of UVI-VTON, VR, and nothing [*F*_(2, 68)_ = 7.299; *p* = 0.006]. *Post-hoc* analysis revealed no significant difference (*p* = 1.000) between the use of UVI-VTON and VR for question 4. However, the use of UVI-VTON showed a significantly higher rating of 0.686 (*p* = 0.0133) than the use of nothing for question 4. The use of VR also exhibited a significantly higher rating of 0.629 (*p* = 0.042) than the use of nothing for question 4.

When conducting home-based exercise training, significant differences were observed in question 5 of SMS among the use of UVI-VTON, VR, and nothing [*F*_(2, 68)_ = 8.279; *p* = 0.003). *Post-hoc* analysis revealed no significant difference (*p* = 0.506) between the use of UVI-VTON and VR for question 5. However, the use of UVI-VTON showed a significantly higher rating of 0.743 (*p* = 0.004) than the use of nothing for question 5. The use of VR also exhibited a significantly higher rating of 0.600 (*p* < 0.039) than the use of nothing for question 5. When conducting home-based exercise training, there were no significant differences in question 6 of CSS among groups using UVI-VTON, VR, and no intervention [*F*_(2, 68)_ = 2.121; *p* = 0.136].

The present study aimed to investigate emotional responses of participants regarding home-based exercise training with or without the use of UVI-VTON or VR. This study is a pioneering effort to develop and apply VTON and VR in home-based exercise training. Understanding impacts of these new methodologies on participants can help us develop and provide more effective exercise programs.

In this study, three measurement criteria were used to evaluate emotional responses of participants, namely PPS, SMS, and CSS. PPS was used to assess perceptions regarding privacy protection. SMS was used to measure confidence and motivation levels of users and CSS was used to evaluate user satisfaction with exercise posture and feedback.

**Figure 6:**
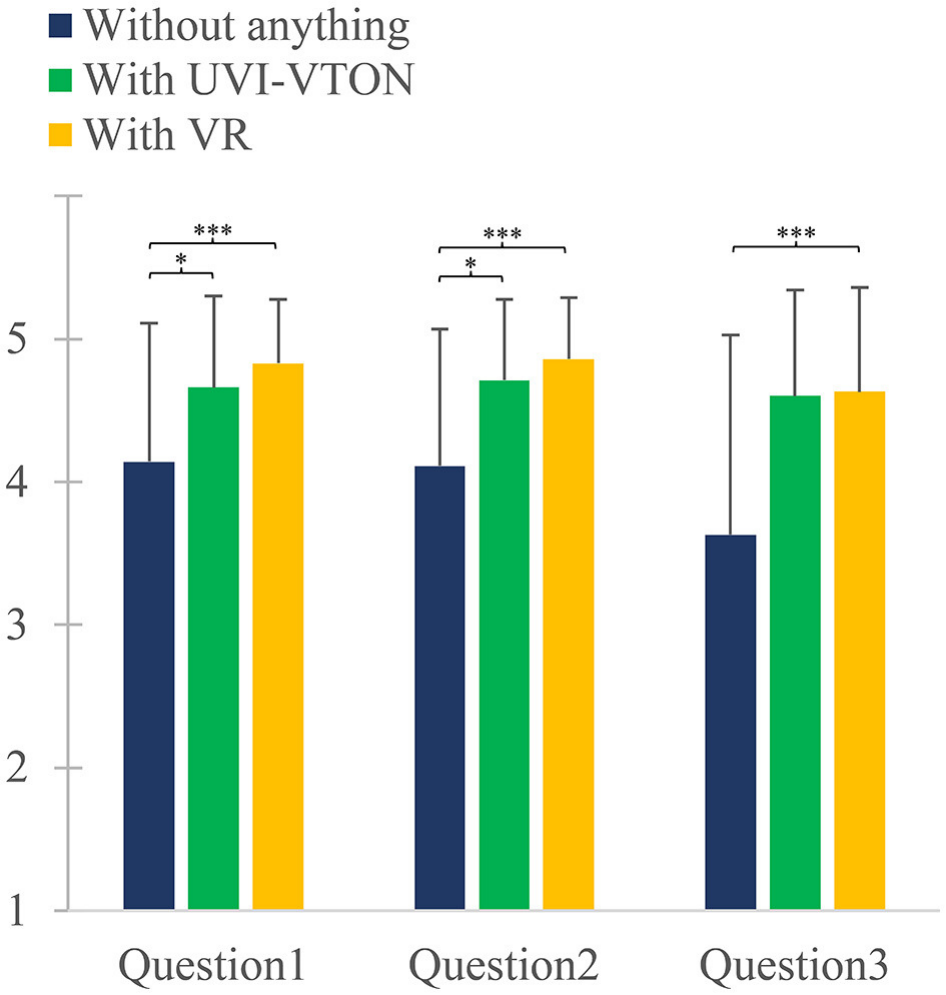
Privacy Protection Scale (PPS) consisting of three questions (Question 1, Question 2, Question 3) for all participants. PPS serves as a tool to assess participants' privacy protection levels based on the presence or absence of UVI-VTON and VR. Vertical error bars represent standard deviation for response for each question. *p < 0.05, ***p < 0.001.

**Figure 7:**
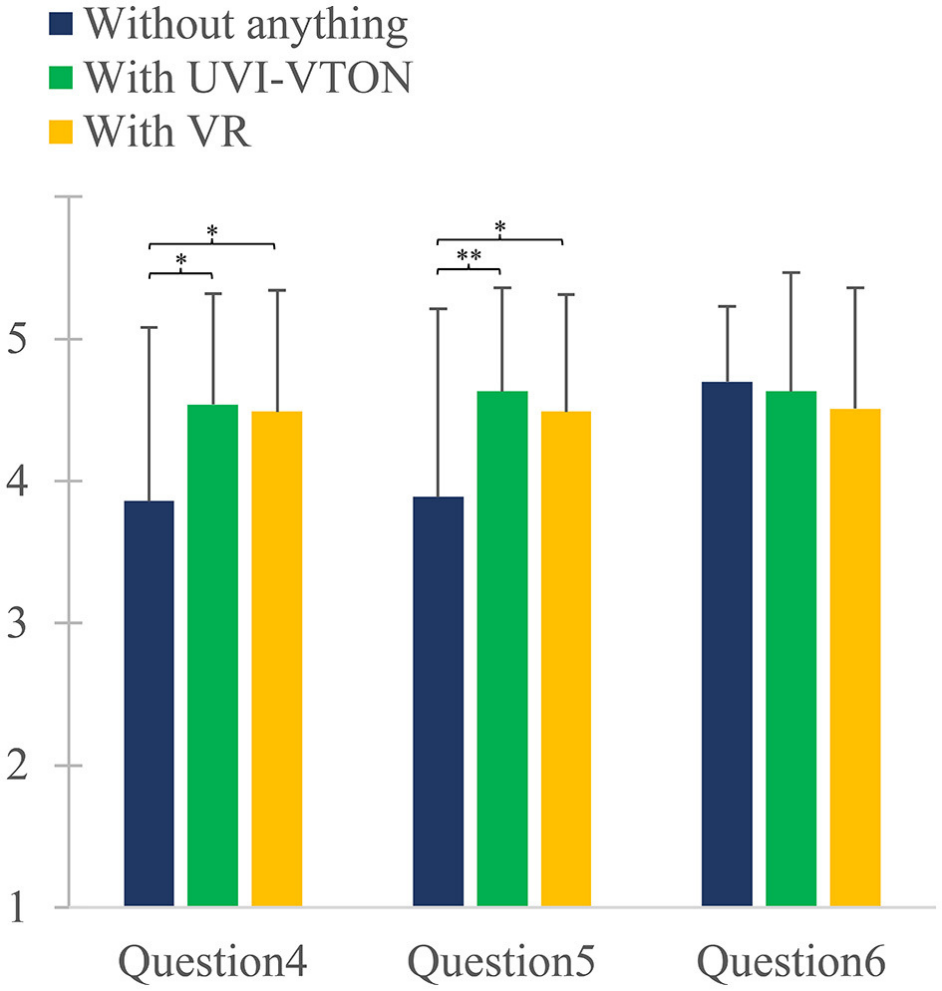
Self-confidence and Motivation Scale (SMS) comprising questions 4 and 5 is employed to assess participants' self-confidence and motivation levels with consideration of the presence or absence of UVI-VTON and VR. Additionally, the Coaching Satisfaction Scale (CSS), comprising a single question (Question 6), is used to gauge participants' coaching satisfaction level, taking into account the presence or absence of UVI-VTON and VR. Vertical error bar represents standard deviation for response for each question. *p < 0.05; **p < 0.005.

As seen in Figure 6, significant differences in PPS were observed when comparing the use of UVI-VTON vs. non-use, indicating that UVI-VTON had a positive impact on privacy protection. Similarly, significant differences in PPS were observed between the use of VR vs. non-use, suggesting that VR could also have a positive impact on privacy protection. These research findings demonstrate that the implementation of UVI-VTON or VR can help alleviate concerns regarding user privacy while enhancing trust in exercise programs. Moreover, results indicate the potential of UVI-VTON or VR to improve user exercise experiences and contribution to personal data protection, thus creating a safer and more comfortable exercise environment. Analysis results also revealed no significant differences in PPS between the use of UVI-VTON and VR. This suggests that both methodologies could elicit similar emotional responses in relation to measured criteria for PPS. While UVI-VTON covers all parts except the face, VR obscures all information captured by the camera. One might expect that the PPS would be significantly higher in VR than UVI-VTON owing to its ability to conceal more than just the face. However, when comparing the use of UVI-VTON vs. the use of VR, the absence of a significant difference in questions 4 and 5, as depicted in Figure 7, implies that concerns regarding personal data exposure primarily stem from the exposure of one's body and background while wearing clothing, excluding the face.

From Figure 7, when comparing the use of UVI-VTON vs. non-use, significant differences were observed for SMS comprising questions 4 and 5. This indicates that UVI-VTON has a positive impact on confidence enhancement and motivation. Similarly, significant differences were observed for SMS when comparing the use of VR vs. non-use. This suggests that VR also has a positive impact on confidence enhancement and motivation. These research findings highlight that engaging in exercise while utilizing UVI-VTON or VR technology provides a means to conceal personal appearance, enabling users to perform their exercises with enhanced self-assurance. This aligns with the goal of improving the exercise environment in the proposed home-based exercise training program, which aims to encourage and motivate users to participate in physical activities. Furthermore, analysis results revealed no significant differences in SMS between the use of UVI-VTON and VR. This suggests that both methodologies can elicit similar emotional responses when measured against the criteria for SMS. Similar to findings from the analysis of PPS responses, these results indicate that methodologies capable of obscuring users' bodies except their face and background have potential to enhance self-confidence and promote exercise participation. Additionally, Figure 7 shows that there are no significant differences among the three cases for CSS comprising question 6. This indicates that there is no qualitative difference in guidance by an exercise coach when compared with delivery of postprocessed exercise videos in home-based exercise training using UVI-VTON and VR against the delivery of raw exercise videos without using these methodologies. This finding suggests the potential of UVI-VTON and VR to replace existing camera-based home-based exercise training programs. These results further demonstrate the potential of UVI-VTON and VR to provide positive experiences for individuals participating in home-based exercise training. UVI-VTON and VR can enhance self-confidence, increase motivation, and strengthen privacy protection, which can in turn improve the quality of exercise experiences and help participants maintain healthy lifestyles by fostering a willingness to continue exercising.

Findings of this study show that applying new methodologies to home-based exercise training can improve emotional responses of participants. Specifically, UVI-VTON and VR have positive impacts on privacy protection, confidence enhancement, and exercise motivation. These findings can help promote exercise training participation in the home environment. Advancements made in the home-based exercise training environment through this research are expected to facilitate greater accessibility for a wider population. Furthermore, this study serves as a crucial indicator for developing methodologies aimed for at enhancing home-based exercise training environment.

## 6 Conclusions

This research aims to help individuals who rely on home-based exercise, encompassing patients, older adults, and individuals in need of courage and motivation to engage in physical activities post COVID-19. This study seeks to enhance home-based exercise training experience with the objective of facilitating participant engagement while alleviating their concerns regarding privacy and emotional burden associated with home-based training. To achieve this, we proposed the use of VR and UVI-VTON. Participants were recruited to engage in home-based exercise training using the proposed approach. A usability survey was administered upon training completion to assess the effectiveness and user experiences. Results demonstrated the potential of the proposed method to improve home-based exercise training and enhance the overall exercise participation experience in terms of privacy protection, enhanced self-confidence, motivation, and real-time coaching instructions. These findings suggest that VR or UVI-VTON can be effectively employed to enhance user experiences in home-based exercise training.

## 7 Limitations

This study has a limitation. Since this study was conducted with a demand group for home training, which was dominated by women, it resulted in an unbalanced sample, which might limit the generalizability of results. Therefore, future studies over an extended period with a larger and more diverse participant base while considering factors such as gender, age, and physical attributes need to be conducted to enhance the reliability and potential applicability of this study's results.

## Data availability statement

The raw data supporting the conclusions of this article will be made available by the authors, without undue reservation.

## Ethics statement

The studies involving humans were approved by Sungkyunkwan University IRB approval number: SKKU 2021-12-014. The studies were conducted in accordance with the local legislation and institutional requirements. The participants provided their written informed consent to participate in this study.

## Author contributions

K-IY: Conceptualization, Data curation, Investigation, Methodology, Project administration, Software, Validation, Writing – original draft, Writing – review & editing. T-SJ: Data curation, Methodology, Software, Writing – review & editing. S-CK: Conceptualization, Project administration, Supervision, Writing – review & editing. S-CL: Conceptualization, Project administration, Supervision, Writing – review & editing.
